# Supplementary material for: The economic burden of non-communicable disease mortality in the South Pacific: Evidence from Fiji
Source: PLoS One. 2020 Jul 23;15(7):e0236068. doi: 10.1371/journal.pone.0236068 (PMC7377477; doi:10.1371/journal.pone.0236068)
Supplement: Attachment — Submitted filename: Response to Reviewers.docx [file pone.0236068.s002.docx]

**Response to Reviewers**

**Comments to the Author**

1. Is the manuscript technically sound, and do the data support the conclusions?

The manuscript must describe a technically sound piece of scientific research with data that supports the conclusions. Experiments must have been conducted rigorously, with appropriate controls, replication, and sample sizes. The conclusions must be drawn appropriately based on the data presented.

Reviewer #1: Partly

Reviewer #2: Yes

**Response**: We have revised the methodology and the methods used that are technically sound. We have used the Autoregressive Distributed Lag (ARDL) bounds test to cointegration approach to draw our conclusion. We have used official secondary data which is compiled by Ministry of Health, Fiji and the World Health Organization. Sample used is appropriate and bounds test to cointegration is standard procedure used to analyse time series data. Revised manuscript is based on Fiji as there no data available for other Pacific Island countries. Revised manuscript is based on long time series data which is only available for Fiji.

2. Has the statistical analysis been performed appropriately and rigorously?

Reviewer #1: N/A

Reviewer #2: No

**Response:** We have revised the methodology. We have used bounds test to cointegration approach. One of the advantages of the ARDL model is that it is very flexible where variables of both I(0) and I(1) status can be used to test for a cointegrating relationship. Secondly, the ARDL approach is an advantage to studies using a small sample size for estimation and forecasting [29]. Thirdly, the ARDL approach gives a consistent and unbiased estimate of the long-run parameters and each variable can have their own different lag-lengths compared to conventional cointegration tests. The fourth advantage of the ARDL approach is that it adequately deals with the problems of autocorrelation and endogeneity and provides unbiased and super-consistent coefficients with valid t-statistics. In sum, there are two steps involved in the ARDL bounds test approach to cointegration. We performed a number of robustness test and found our result is consistent. We carried out several diagnostic test and our result suffice all classical assumption.

3. Have the authors made all data underlying the findings in their manuscript fully available?

The [PLOS Data policy](http://www.plosone.org/static/policies.action#sharing) requires authors to make all data underlying the findings described in their manuscript fully available without restriction, with rare exception (please refer to the Data Availability Statement in the manuscript PDF file). The data should be provided as part of the manuscript or its supporting information, or deposited to a public repository. For example, in addition to summary statistics, the data points behind means, medians and variance measures should be available. If there are restrictions on publicly sharing data—e.g. participant privacy or use of data from a third party—those must be specified.

Reviewer #1: No

Reviewer #2: Yes

**Response:** We have uploaded all the data used in the analysis.

4. Is the manuscript presented in an intelligible fashion and written in standard English?

PLOS ONE does not copyedit accepted manuscripts, so the language in submitted articles must be clear, correct, and unambiguous. Any typographical or grammatical errors should be corrected at revision, so please note any specific errors here.

Reviewer #1: Yes

Reviewer #2: Yes

**Response:** Manuscript presented and written in standard English.

5. Review Comments to the Author

Please use the space provided to explain your answers to the questions above. You may also include additional comments for the author, including concerns about dual publication, research ethics, or publication ethics. (Please upload your review as an attachment if it exceeds 20,000 characters)

Reviewer #1: Results have to organised in a better way and figures should be more presentable. Discussion should be brief and to the point. Conclusion does not reflect discussion, it should be rewritten. Some of the references are not organised properly.

**Response:** We have rewritten all our results and discussion. Now it is more presentable, clear and easy to follow. We have rewritten our conclusion and policy implications and now it reflects the findings and discussion.

Reviewer #2: Health economics is a growing field that push the universal health coverage agenda forward for improving health coverage, expanding services, and providing financial-risk protection are all necessary to optimize the population health. Chronic non-communicable disease (NCD) is the big issue in low and middle income countries which really need to focus on the building health systems that integrate complex, multidisciplinary interventions to reduce the economical burden.
This study is focused on estimate the net percentage value loss of non-health GDP, and indirect cost of Non-communicable disease related mortality among the working age group of eight small Pacific Island countries (PIC) in 2017.Using human capital approach methodology with non-health GDP per capita, author emphasize that NCD related premature death in working age population creates a potential economical burden for the PICs.
In most of the countries, the premature mortality due to cardiovascular disease and in working ages from 15 until the retirement age. Also, this study has recommended intervention to reduce the prevalence of NCD related that all small PICs should reduce the prevalence of NCD related premature death.
This manuscript well written and describe the economical burden for the small Pacific Islands countries and also the interventions to reduce.
The following comments should be resolved:
(1) Which statistical analysis was used in this study to claim that NCD-related death in the working age group create a significant economic burden in the PIC?

**Response:** Our revised manuscript is extensively rewritten based on the comments and suggestion of two reviewers. In the revised manuscript, we have used bounds test to cointegration approach, it is standard procedure used to analyse factors affecting economic output. We carried out a several robustness test and find out result is consistent. Revised manuscript is based on Fiji as there no data available for other Pacific Island countries. Revised manuscript is based on long time series data which is only available for Fiji.

(2) Figure 1, bar diagram shows that percentage of total death is increased in NCD group in different group, but it is not clear that it is significant or not as no statistical analysis was shown.

**Response:** All results and graph figures are revised based on our revised findings. We have used ARDL bounds test to analyse our data. We carried out a number of diagnostic test and find our result is reliable.

(3) Same in case of figure 3.

**Response:** All our analysis is based on ARDL bounds testing technique. However, this comment is no longer relevant in revised manuscript.

6. PLOS authors have the option to publish the peer review history of their article ([what does this mean?](https://journals.plos.org/plosone/s/editorial-and-peer-review-process#loc-peer-review-history)). If published, this will include your full peer review and any attached files.


If you choose “no”, your identity will remain anonymous but your review may still be made public.


**Do you want your identity to be public for this peer review?** For information about this choice, including consent withdrawal, please see our [Privacy Policy](https://www.plos.org/privacy-policy).

Reviewer #1: No

Reviewer #2: No


[NOTE: If reviewer comments were submitted as an attachment file, they will be attached to this email and accessible via the submission site. Please log into your account, locate the manuscript record, and check for the action link "View Attachments". If this link does not appear, there are no attachment files to be viewed.]


While revising your submission, please upload your figure files to the Preflight Analysis and Conversion Engine (PACE) digital diagnostic tool, <https://pacev2.apexcovantage.com/>. PACE helps ensure that figures meet PLOS requirements. To use PACE, you must first register as a user. Registration is free. Then, login and navigate to the UPLOAD tab, where you will find detailed instructions on how to use the tool. If you encounter any issues or have any questions when using PACE, please email us at [figures@plos.org](mailto:figures@plos.org). Please note that Supporting Information files do not need this step.
